# Supplementary material for: Two-dimensional fetal speckle tracking; a learning curve study for offline strain analysis
Source: PLoS One. 2024 Nov 18;19(11):e0310307. doi: 10.1371/journal.pone.0310307 (PMC11573155; doi:10.1371/journal.pone.0310307)

**Appendix:** Bland Altman plots

Plots of LV-GLS difference between expert and fellow over four consecutive groups of 25 fetal heart clips


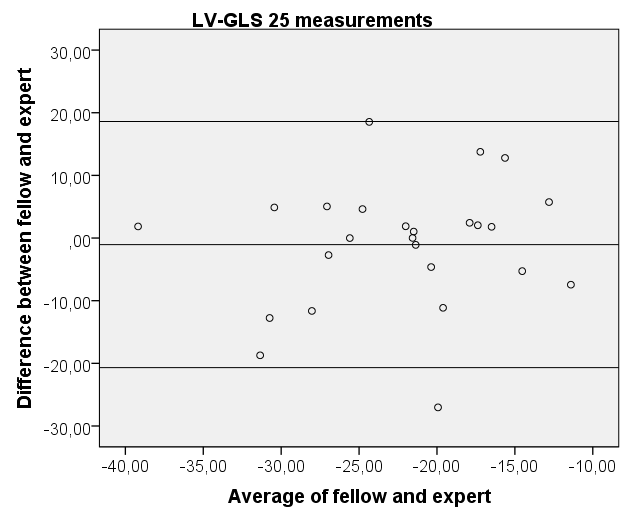

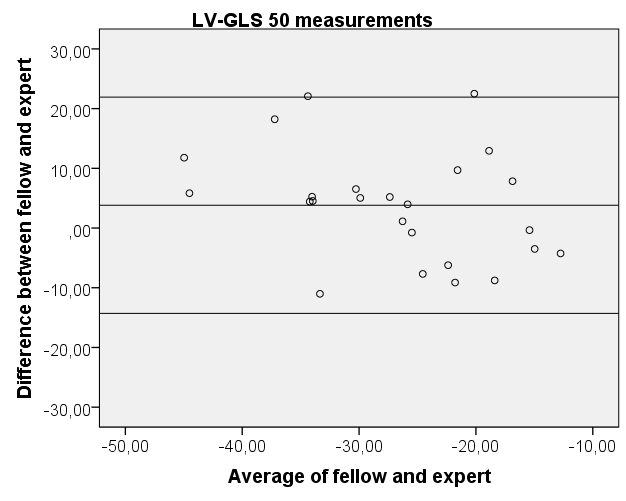


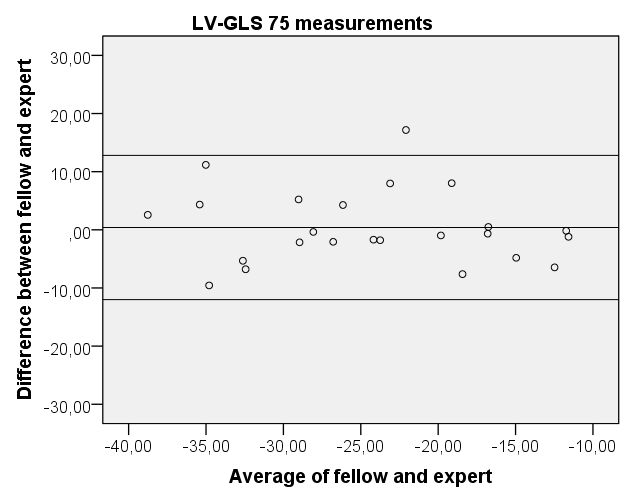

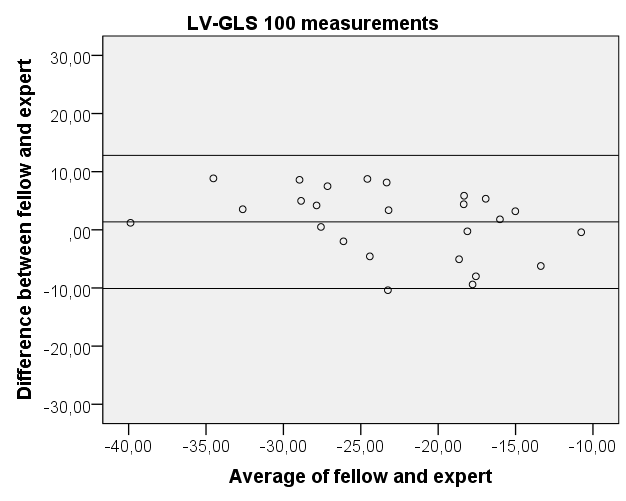


Plots of LV-GLS difference between expert and resident over four consecutive groups of 25 fetal heart clips


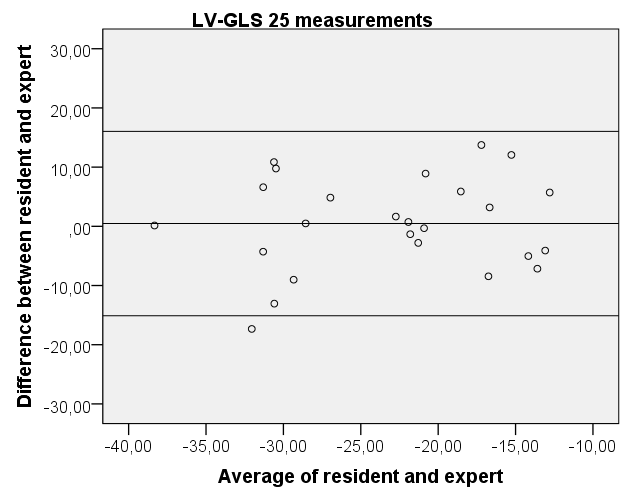

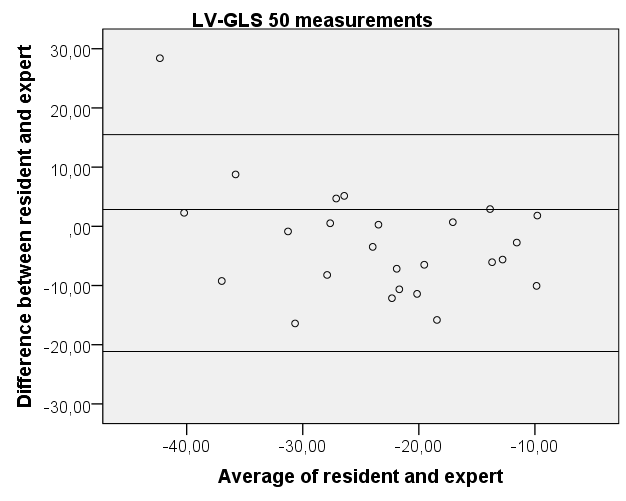


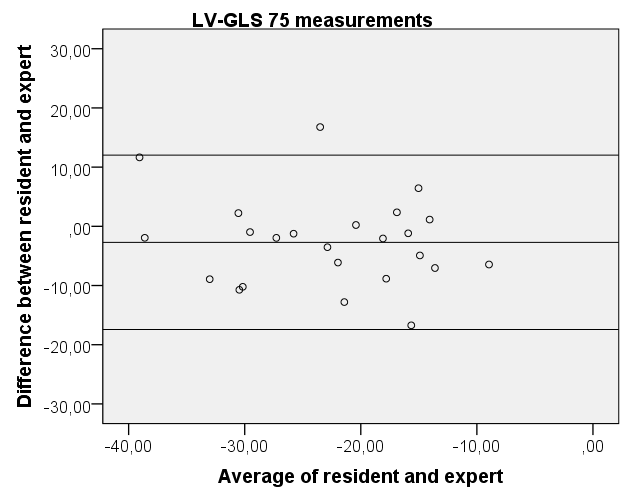

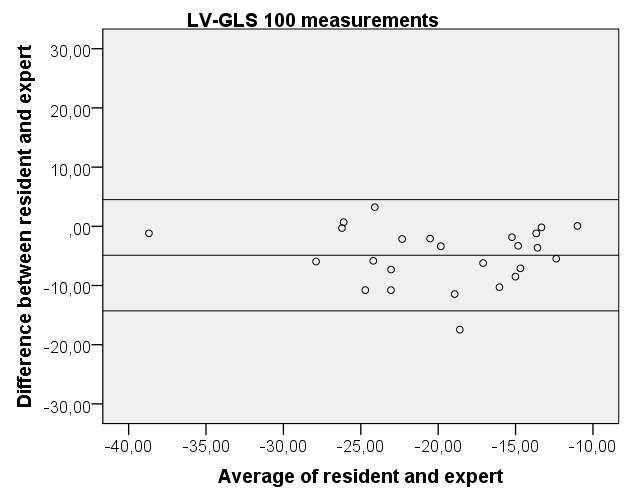


Plots of LV-GLS difference between expert and student over four consecutive groups of 25 fetal heart clips


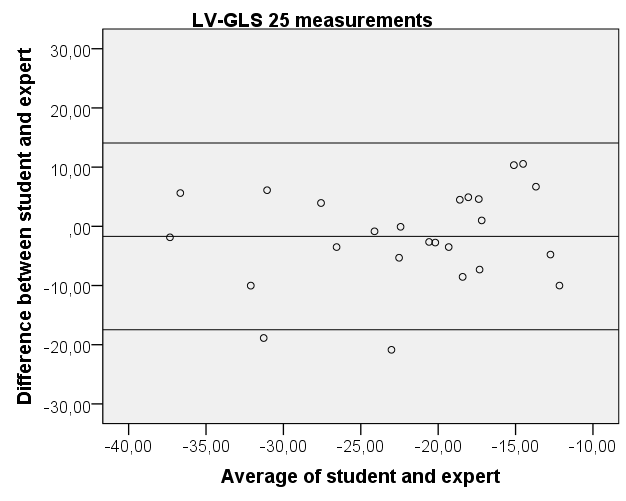

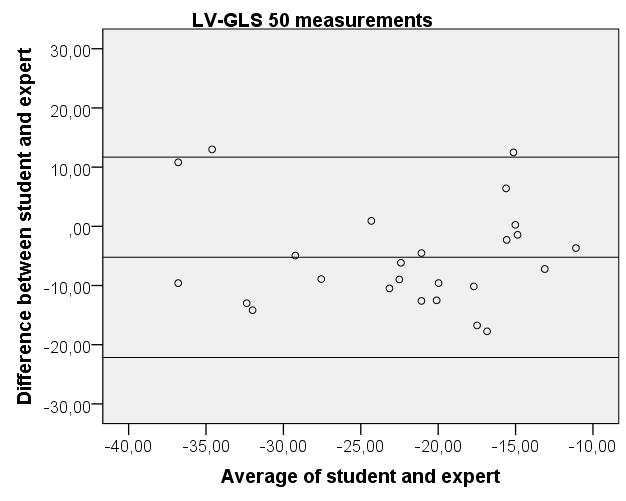


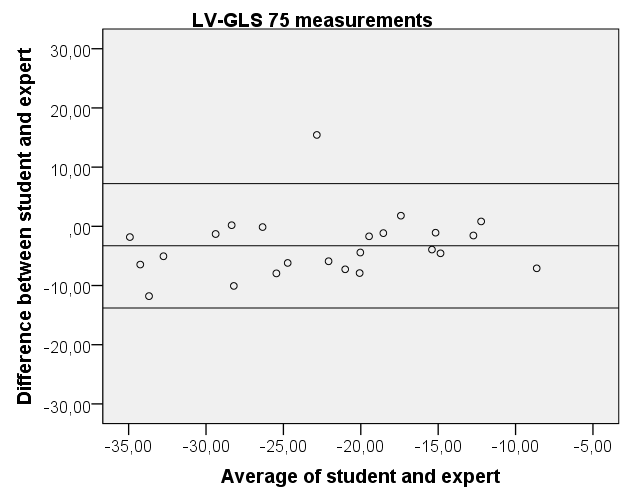

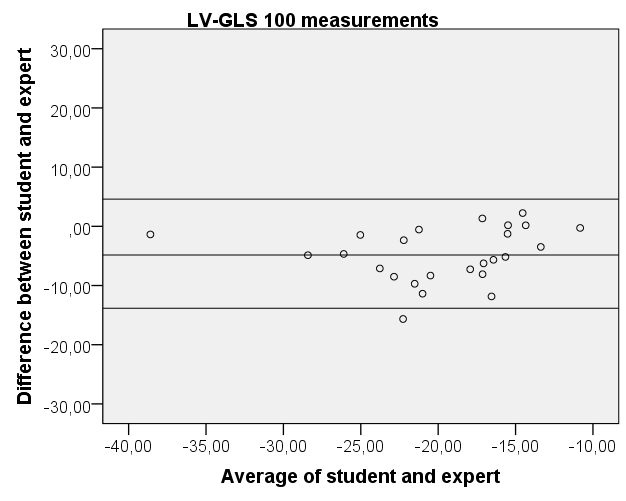


Plots of RV-GLS difference between expert and fellow over four consecutive groups of 25 fetal heart clips


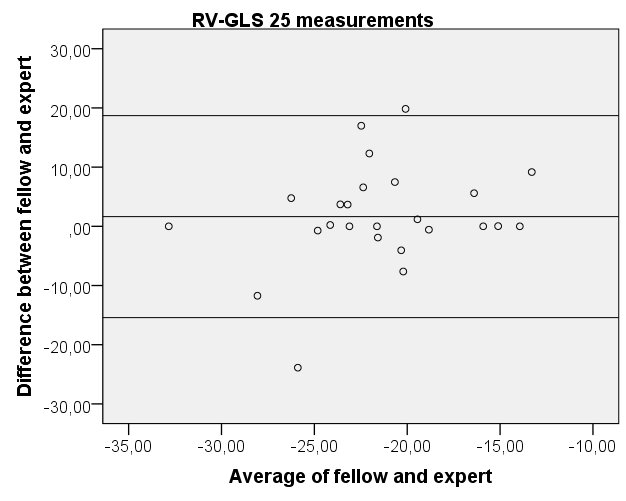

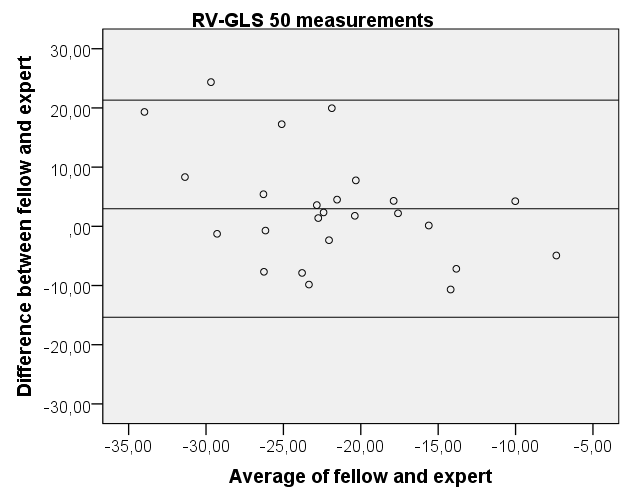


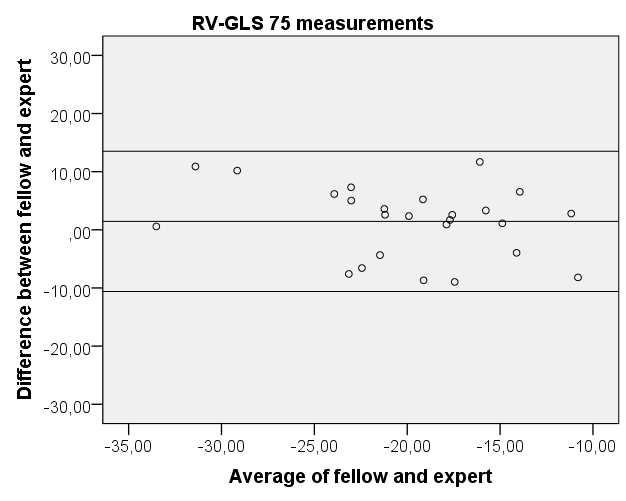

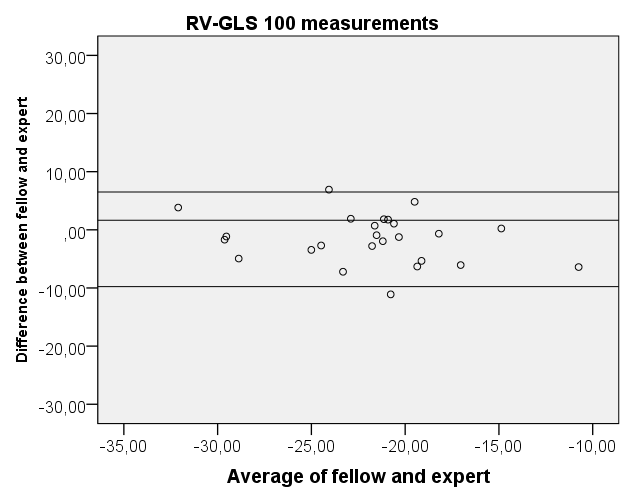


Plots of RV-GLS difference between expert and resident over four consecutive groups of 25 fetal heart clips


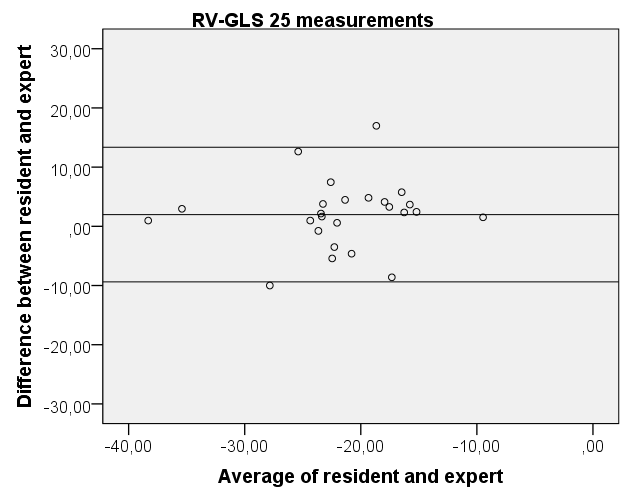

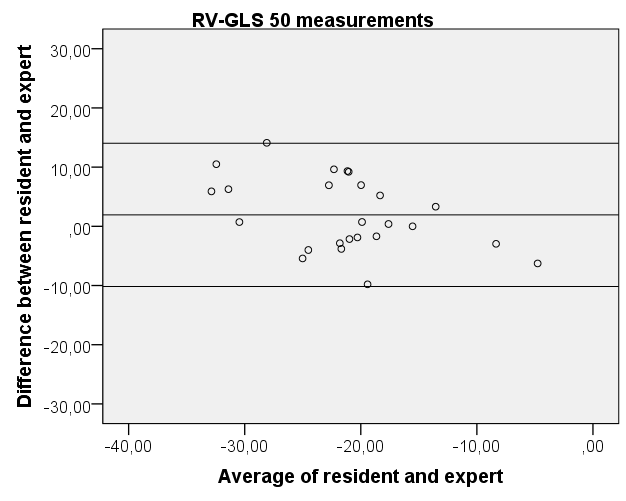


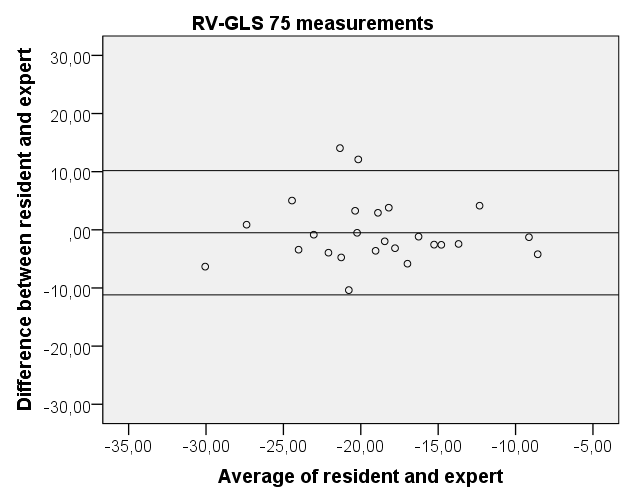

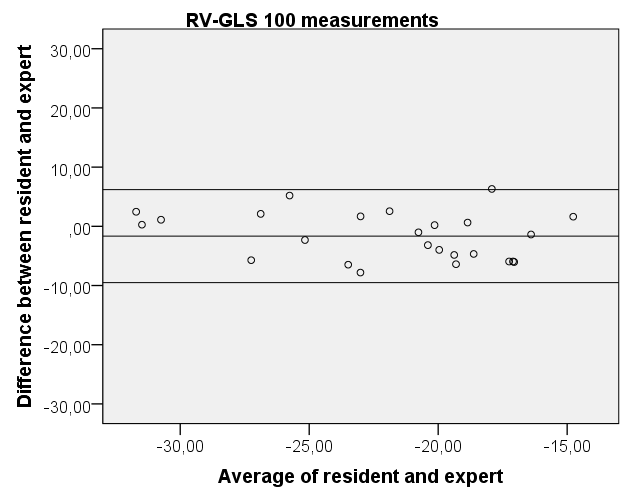


Plots of RV-GLS difference between expert and student over four consecutive groups of 25 fetal heart clips


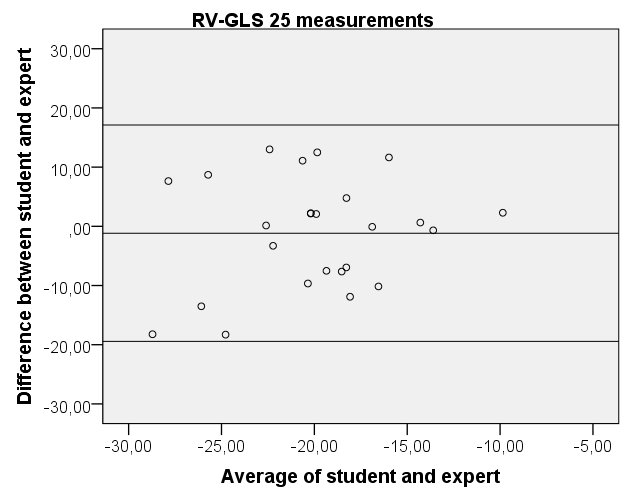

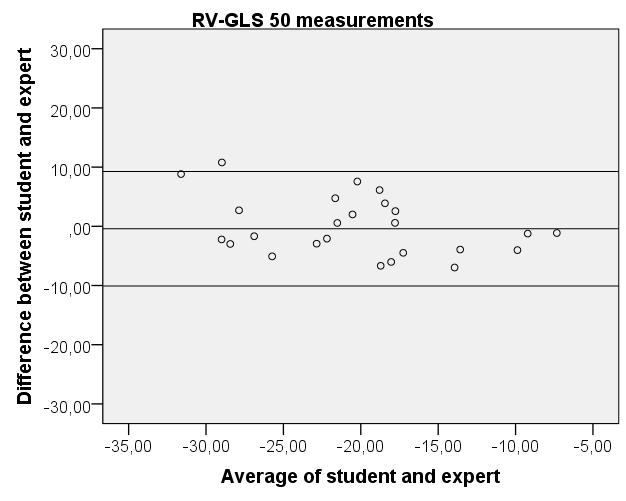


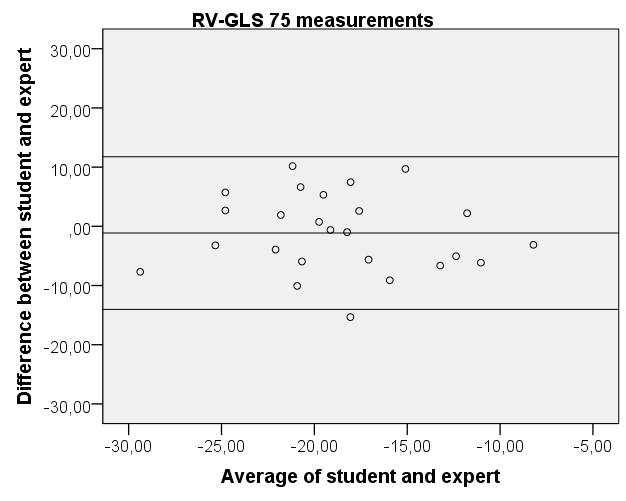

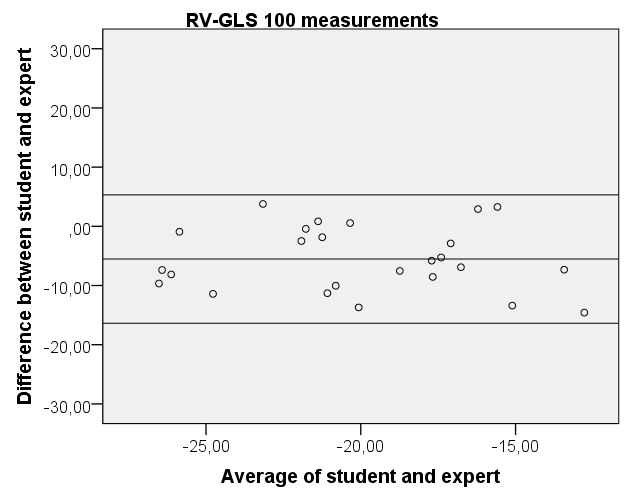

Supplement: S1 Appendix — (DOCX) [file pone.0310307.s001.docx]
