# Supplementary material for: Two-dimensional fetal speckle tracking; a learning curve study for offline strain analysis
Source: PLoS One. 2024 Nov 18;19(11):e0310307. doi: 10.1371/journal.pone.0310307 (PMC11573155; doi:10.1371/journal.pone.0310307)
Supplement: Attachment — Submitted filename: PONE-D-23-44042.docx [file pone.0310307.s003.docx]

PONE-D-23-44042 5.6.2024

Dear authors,

It is interesting to compare trainees' learning curves to those of an expert in fetal echocardiography, especially fetal global longitudinal strain. In general, this is a very well-written article and a good idea, but one uncorrectable flaw is present in the reviewer's opinion.

An article should inform us of some new ideas, and statistical methods should be used to show how it is replicable in a different population (in this case, trainees). Since there were only three probands compared to one expert, we cannot draw any conclusions and accept any recommendation based on these data even less. The reviewer suggests increasing the probands by at least ten in a group and then comparing the group variability to correct for inter-individual variability of the learning curves. After completion of the testing rounds, there may be an add-on evaluation to correct for intra-individual variability, which can be found even in experts. Then, the reviewer will find this manuscript worth publishing in Plos One and encourage the authors to resubmit it.
