# Supplementary material for: Two-dimensional fetal speckle tracking; a learning curve study for offline strain analysis
Source: PLoS One. 2024 Nov 18;19(11):e0310307. doi: 10.1371/journal.pone.0310307 (PMC11573155; doi:10.1371/journal.pone.0310307)
Supplement: Attachment — Submitted filename: Response to Reviewers.docx [file pone.0310307.s004.docx]

**Rebuttal letter**

Academic Editor

Professor Kumaradevan Punithakumer
c/o PLOS ONE

Utrecht, The Netherlands, 1^st^ of August 2024

Dear Professor Kumaradevan Punithakumer,

On behalf of all the authors, I hereby kindly resubmit our study entitled “Fetal strain analysis with two-dimensional speckle tracking; a learning curve study” to PLOS ONE. We greatly appreciate your willingness to reconsider our revised manuscript where we have addressed the reviewers’ criticisms. We sincerely thank the reviewers for their valuable feedback, which has enhanced the quality of our manuscript. Please find our revision notes and responses to the reviewers below. We have also added the revised manuscript, which incorporates their feedback

This research article represents the first study on the learning curve for fetal strain analysis using two-dimensional speckle tracking echocardiography. A clear learning effect was demonstrated for three trainees new to the technique. The study also discusses suggestions to improve speckle tracking for fetal application. A follow-up learning curve study with at least 10 trainees per group, as suggested by reviewer 3, should be performed after improvement of the technique.

We confirm that this work is original and has not been published, nor is it currently under consideration for publication elsewhere. For this study, no funding was received and we have no conflicts of interest to disclose. All authors confirmed to share responsibility and approved the manuscript as submitted.

Please address all correspondence concerning this manuscript to me at: [chantelle.de.vet@mmc.nl](mailto:chantelle.de.vet@mmc.nl).

Thank you for your consideration of this manuscript.

On behalf of all the authors, Yours sincerely,

Chantelle de Vet, MD, PhD, resident gynaecology

Thomas Nichting, MD, PhD, resident gynaecology

Annemarie Fransen, MD, PhD, fellow perinatology

Daisy van der Woude, MD, PhD, consultant obstetrician

Myrthe van der Ven, MSc, PhD, technical physician

Zoé van Lier, BSc, medical student

Richard Post, Ir, PhD, statistician

Guid Oei, MD, PhD, consultant obstetrician, Professor Signal Processing Systems group

Noortje van Oostrum, MD, PhD, consultant obstetrician

Judith van Laar, MD, PhD, consultant obstetrician, Associate Professor Signal Processing Systems group

**Journal requirements:**

When submitting your revision, we need you to address these additional requirements.

1. Please ensure that your manuscript meets PLOS ONE's style requirements, including those for file naming. The PLOS ONE style templates can be found at
<https://journals.plos.org/plosone/s/file?id=wjVg/PLOSOne_formatting_sample_main_body.pdf> and
<https://journals.plos.org/plosone/s/file?id=ba62/PLOSOne_formatting_sample_title_authors_affiliations.pdf>

**Response:**  I have amended the manuscript accordingly.

2. Please note that PLOS ONE has specific guidelines on code sharing for submissions in which author-generated code underpins the findings in the manuscript. In these cases, all author-generated code must be made available without restrictions upon publication of the work. Please review our guidelines at <https://journals.plos.org/plosone/s/materials-and-software-sharing#loc-sharing-code> and ensure that your code is shared in a way that follows best practice and facilitates reproducibility and reuse.

**Response:** I have added the SAS codes for the analysis and the R codes for the figures.

3. We note that Figure 1 in your submission contain [map/satellite] images which may be copyrighted. All PLOS content is published under the Creative Commons Attribution License (CC BY 4.0), which means that the manuscript, images, and Supporting Information files will be freely available online, and any third party is permitted to access, download, copy, distribute, and use these materials in any way, even commercially, with proper attribution. For these reasons, we cannot publish previously copyrighted maps or satellite images created using proprietary data, such as Google software (Google Maps, Street View, and Earth). For more information, see our copyright guidelines: <http://journals.plos.org/plosone/s/licenses-and-copyright>.
 
We require you to either (1) present written permission from the copyright holder to publish these figures specifically under the CC BY 4.0 license, or (2) remove the figures from your submission:
 
1. You may seek permission from the original copyright holder of Figure 1 to publish the content specifically under the CC BY 4.0 license.  
 
We recommend that you contact the original copyright holder with the Content Permission Form (<http://journals.plos.org/plosone/s/file?id=7c09/content-permission-form.pdf>) and the following text:
“I request permission for the open-access journal PLOS ONE to publish XXX under the Creative Commons Attribution License (CCAL) CC BY 4.0 (<http://creativecommons.org/licenses/by/4.0/>). Please be aware that this license allows unrestricted use and distribution, even commercially, by third parties. Please reply and provide explicit written permission to publish XXX under a CC BY license and complete the attached form.”
 
Please upload the completed Content Permission Form or other proof of granted permissions as an ""Other"" file with your submission.
 
In the figure caption of the copyrighted figure, please include the following text: “Reprinted from [ref] under a CC BY license, with permission from [name of publisher], original copyright [original copyright year].”
 
2. If you are unable to obtain permission from the original copyright holder to publish these figures under the CC BY 4.0 license or if the copyright holder’s requirements are incompatible with the CC BY 4.0 license, please either i) remove the figure or ii) supply a replacement figure that complies with the CC BY 4.0 license. Please check copyright information on all replacement figures and update the figure caption with source information. If applicable, please specify in the figure caption text when a figure is similar but not identical to the original image and is therefore for illustrative purposes only.
The following resources for replacing copyrighted map figures may be helpful:
 
USGS National Map Viewer (public domain): <http://viewer.nationalmap.gov/viewer/>
The Gateway to Astronaut Photography of Earth (public domain): <http://eol.jsc.nasa.gov/sseop/clickmap/>
Maps at the CIA (public domain): <https://www.cia.gov/library/publications/the-world-factbook/index.html> and <https://www.cia.gov/library/publications/cia-maps-publications/index.html>
NASA Earth Observatory (public domain): <http://earthobservatory.nasa.gov/>
Landsat: <http://landsat.visibleearth.nasa.gov/>
USGS EROS (Earth Resources Observatory and Science (EROS) Center) (public domain): [http://eros.usgs.gov/#](http://eros.usgs.gov/)
Natural Earth (public domain): <http://www.naturalearthdata.com/>

**Response:** Figure 1 is a screenshot of a strain analysis performed by one of our co-authors (the expert dr. N van Oostrum). The Figure has not been published before in any of our previous articles. I have been in contact with a journal staff member, Teresa Diviacchi, Peer Review Operations Specialist. She has reviewed Figure 1 and confirmed there are no copyright concerns. Please see our e-mail correspondence below.

**Van:** plosone <plosone@plos.org>
**Verzonden:** woensdag 31 juli 2024 20:30
**Aan:** Vet, Chantelle de <Chantelle.de.Vet@mmc.nl>
**Onderwerp:** RE: PLOS ONE Decision: Revision required [PONE-D-23-44042] - Question on copyright Fig 1

Dear Dr. de Vet,

Thank you for your query.

I've reviewed Fig 1 and agree, this shouldn't pose any copyright concerns. When submitting your revised submission, please respond to journal question 3 by clarifying these are images of tests run by you, the authors, and that a journal staff member has reviewed the images and confirmed there are no copyright concerns.

Let me know if you have any further questions. We look forward to receiving you revised manuscript.

Kind regards,
Teresa

Teresa Diviacchi
Peer Review Operations Specialist

PLOS ONE | plosone@plos.org
Empowering researchers to transform science
1265 Battery Street, Suite 200 San Francisco, CA 94111

Case Number: 08583403
ref:!00DU00Ifis.!500PM0NIIkP:ref


--------------- Original Message ---------------
**From:** Vet, Chantelle de [chantelle.de.vet@mmc.nl]
**Sent:** 7/25/2024, 10:07 AM
**To:** plosone@plos.org; figures@plos.org
**Subject:** RE: PLOS ONE Decision: Revision required [PONE-D-23-44042] - [EMID:eaf5756556d5b86e] - Question on copyright Fig 1

Dear Editor or Editorial assistant,

I have a question about the copyright status of Figure 1 that is addressed in point 3 of the Journal Requirements. This figure is a screenshot of one of our own analyses conducted by the expert (one of the co-authors, Dr. van Oostrum). This figure has not been published in any of our previous articles.

Can we proceed without obtaining additional copyright clearance, since the analysis was conducted by one of our co-authors? Or should we seek permission from the manufacturer, TomTec, for the use of this figure?  Or the co-author expert? We want to ensure we are in compliance with your publication policies.

Thank you for your assistance.

Kind regards, Met vriendelijke groet,

**Chantelle de Vet - Arts onderzoeker Obstetrie en Gynaecologie**

Máxima Medisch Centrum Veldhoven, De Run 4600, 5504 DB Veldhoven

Postbus 7777, 5500 MB Veldhoven

**Response to reviewers**

**Comments to the Author**

1. Is the manuscript technically sound, and do the data support the conclusions?

The manuscript must describe a technically sound piece of scientific research with data that supports the conclusions. Experiments must have been conducted rigorously, with appropriate controls, replication, and sample sizes. The conclusions must be drawn appropriately based on the data presented.

Reviewer #1: Yes

Reviewer #2: Yes

Reviewer #3: No

Reviewer #4: Yes

2. Has the statistical analysis been performed appropriately and rigorously?

Reviewer #1: Yes

Reviewer #2: Yes

Reviewer #3: N/A

Reviewer #4: Yes

3. Have the authors made all data underlying the findings in their manuscript fully available?

The [PLOS Data policy](http://www.plosone.org/static/policies.action#sharing) requires authors to make all data underlying the findings described in their manuscript fully available without restriction, with rare exception (please refer to the Data Availability Statement in the manuscript PDF file). The data should be provided as part of the manuscript or its supporting information, or deposited to a public repository. For example, in addition to summary statistics, the data points behind means, medians and variance measures should be available. If there are restrictions on publicly sharing data—e.g. participant privacy or use of data from a third party—those must be specified.

Reviewer #1: Yes

Reviewer #2: Yes

Reviewer #3: Yes

Reviewer #4: Yes

4. Is the manuscript presented in an intelligible fashion and written in standard English?

PLOS ONE does not copyedit accepted manuscripts, so the language in submitted articles must be clear, correct, and unambiguous. Any typographical or grammatical errors should be corrected at revision, so please note any specific errors here.

Reviewer #1: Yes

Reviewer #2: Yes

Reviewer #3: Yes

Reviewer #4: Yes

**5. Review Comments to the Author**

Please use the space provided to explain your answers to the questions above. You may also include additional comments for the author, including concerns about dual publication, research ethics, or publication ethics. (Please upload your review as an attachment if it exceeds 20,000 characters)

**Reviewer #1:** 

I would suggest that in the results chapter of the abstract as the only revision to remove the numerical values.

The introduction is well written and concise.

Material and method adequately described. In figure 1 I suggest you to improve the image quality because it is very difficult to discern the obtained values and end-diastolic moments in M-mode.

The results are consistent and support the conclusions. As a small comment from what it seems from the results for examiners without domain expertise it would probably require more than 100 examinations analyzed to adequately interpret the speckle tracking volumes.

The discussions are consistent and point out very well all the intricacies of the method and the learning curve of this method.

As said the conclusions are adequate and supported by the results of the study.

**Response to Reviewer #1**

Thank you for your sincere feedback.

In the results section of the abstract, we aimed to summarize the learning curves of the trainees. We chose to retain the numerical values because the learning curves are individual and differ for all three trainees.

We have made an effort to improve the quality of Figure 1 and used the journal recommended Preflight Analysis and Conversion Engine (PACE) digital diagnostic tool which ensures that figures meet PLOS requirements.

We fully agree with your comment; if one strives for excellent consistency of the trainee compared to the expert (according to the adult learning curve paper), there are more than 100 training measurements needed. Alternatively, the speckle tracking technique should be further improved to reduce user dependency. Then, the learning curve of strain analysis with speckle tracking could be re-investigated and there are may be fewer training measurements needed. We have amended the Discussion – page 19-20: *The second explanation could be that the training might have been too minimal to achieve an excellent learning curve compared to adult GLS learning curves, suggesting that more training measurements are required for fetal GLS analysis. The training in this study was based on the method by Chan et al. for adult LV-GLS analysis involving, two supervised measurements followed by 100 exercises using 2D-STE. To directly promote error correction, access to informative feedback by an experienced user of fetal speckle tracking on buttonology and offline analysis was available upon request throughout the 100 measurements. To ensure successful training for new users, it is suggested to incorporate the elements of deliberate practice^26,27^. The duration of learning may vary for each individual. Training should be continued until the trainees reach the expert level rather than being based on a fixed number of training measurements ^26,28^. The speckle tracking technique should be further improved to reduce user-dependency. Once improvements are made, the learning curve of strain analysis with speckle tracking could be re-investigated, potentially requiring fewer training measurements. In addition, this study only investigated individual learning curves. To determine the number of training measurements needed before strain can be reported independently, the learning curve should be assessed in a larger group of at least ten trainees after the technique is further improved.*

**Reviewer #2:**

The Authors analyzed the reproducibility and learning curve of fetal 2D-STE.
Their results highlight the importance of an adequate learning curve for avoiding mistakes in both the acquisition and the interpretation of fetal 2D-STE.
The Authors also suggested some strategies to minimize the inter-observer variability, such as the ECG synchronization and minimizing the manual adjustment of the tracking lines by refining the automatic segmentation within the 2D-STE software.
I agree with the Authors' findings.
I have only a minor suggestion for the Authors.
In the Limitations section, the Authors could summarize the main limitations of strain echocardiographic imaging, particularly its dependence on:
1) good image quality;
2) frame rates (generally, no less than 80 fps for fetal 2D-STE); the tracking quality becomes reduced when the frame rate is too low;
3) the operator’s experience;
4) loading conditions (pre- and after-load);
5) the ultrasound system employed for the analysis (inter-vendor variability).
The Authors could cite the following references: PMID: 25762560, PMID: 28528162 and PMID: 36142856.

**Response to Reviewer #2**

Thank you for your valuable feedback on the limitations section and the pointed out references. We have amended the text accordingly (See page 20-21 of the Revised manuscript with track changes):

Discussion – page 20-21: *This is a strength and limitation of the study because acquisition settings have an important impact on the measurements. These settings include frame rates, and image quality and resolution which are dependent on the operator’s experience and ultrasound system and transducer used^32-34^. For this study, the same image acquisition settings and same operator were used. If image acquisition variability by different trainees was included in the learning curve, this would complicate the determination of whether the variation in GLS measurements between trainees was due to image acquisition or offline GLS analysis.*

**Reviewer #3:**

Dear authors,
It is interesting to compare trainees' learning curves to those of an expert in fetal echocardiography, especially fetal global longitudinal strain. In general, this is a very well-written article and a good idea, but one uncorrectable flaw is present in the reviewer's opinion.
An article should inform us of some new ideas, and statistical methods should be used to show how it is replicable in a different population (in this case, trainees). Since there were only three probands compared to one expert, we cannot draw any conclusions and accept any recommendation based on these data even less. The reviewer suggests increasing the probands by at least ten in a group and then comparing the group variability to correct for inter-individual variability of the learning curves. After completion of the testing rounds, there may be an add-on evaluation to correct for intra-individual variability, which can be found even in experts. Then, the reviewer will find this manuscript worth publishing in Plos One and encourage the authors to resubmit it.

**Response to Reviewer #3**

Thank you for your feedback. We agree with the Reviewer that more trainees per group would be of value in a follow-up study to determine the number of training measurements needed before one can report strain values independently. This is only the first study examining the learning curve of fetal strain analysis with two-dimensional speckle tracking. The aim of this study was to investigate the individual learning curves of strain analysis by three trainees with different levels of fetal echocardiographic experience.

Speckle tracking is considered worldwide by experts to be a promising technique to measure fetal cardiac function in certain pregnancy diseases and congenital heart defects and there are many papers on speckle tracking. However, the authors have some concerns according to this technique; there are only a few papers on reproducibility and there is no literature on the learning curve of users new to speckle tracking. This paper highlights that there is a clear learning effect for strain analysis; the trainee experienced with fetal heart ultrasound (without any experience with speckle tracking) showing the closest resemblance to the expert. However, this paper also highlights that the technique is still limited by the relatively low inter-observer reproducibility. The authors believe that the technique has to be improved for fetal application first, before it can be implemented in clinical practice. The authors give recommendations on how to improve the technique and consistency among centers using speckle tracking. Before implementation, it is also needed to examine the number of training measurements needed for a new user before independently report strain values, according to the feedback of Reviewer 3. The authors have amended the text in the Discussion accordingly:

Discussion – page 20: *In addition, this study only investigated individual learning curves. To determine the number of training measurements needed before strain can be reported independently, the learning curve should be assessed in a larger group of at least ten trainees after the technique is further improved.*

Discussion – page 22: *However, the learning curves were limited by the relatively low inter-observer reproducibility of speckle tracking in fetuses compared to adults and should be reexamined after the reproducibility has improved. Then, the number of measurements needed to achieve expert competency for fetal GLS analysis using 2D-STE, should be examined in a group of at least ten trainees experienced with fetal heart ultrasound.*

**Reviewer #4:**

Fetal echocardiography, until recently, was used exclusively to identify cardiac malformations. In recent years, however, special attention has been paid to the evaluation of the cardiac ventricular function in the fetus. The assessment of fetal cardiac function is challenging, given the small size and motion of the fetal heart, the moving fetus and the negative influence of high maternal body mass index
Two-dimensional speckle tracking echocardiography (2D-STE) represents an innovative parameter for the quantification of ventricular function which has gained ground in recent years. The use of this method during the fetal period is developing. That is why I consider that such a fetal study is welcomed.
1. The introduction is of appropriate length. On the other hand, from my point of view, the information provided by the authors is rather general, I would prefer something more specific about fetal 2D-STE.
2. The chapter on the method used is very explicit and well written. I would like to congratulate the authors for the complex but at the same time easy-to-follow way of this chapter.
3. The obtained results bring important information in the field of fetal 2D-STE that can be used to facilitate the implementation of this method.
4. The statistical analysis used is appropriate for this research.
5. Although there are few studies that address this topic, the discussions in the article are well written.
6. The conclusions are relevant to this article.
7. Tables and figures are clear and informative.

In conclusion, I congratulate the authors for the work done, for the clarity of this research but also for the new information brought in the field of fetal 2D-STE

**Response to Reviewer #4**

Thank you for your kind words and feedback. The authors agree with the reviewer that speckle tracking echocardiography is a promising technique for measuring fetal cardiac function, but there are still challenges to overcome within this technique that have to be examined.

In response to your comment 1, we have specified the example coarctation of the aorta. The authors believe that speckle tracking can significantly improve the detection of this congenital heart disease. Please see our amendments in the Introduction (page 4): *Fetal GLS measurements may also be of value in the diagnosis and follow-up of congenital heart defects, for example in coarctation of the aorta where the prenatal detection rate is low, while the false positive rate is relatively high. Speckle tracking values are shown to increase the detection of coarctation when used complementary to conventional parameters^10–12^. Prenatal diagnosis of these defects enables timely, adequate treatment and therefore reduces morbidity and mortality^13,14^.*
